# Robust Mercury Methylation across Diverse Methanogenic Archaea

**DOI:** 10.1128/mBio.02403-17

**Published:** 2018-04-10

**Authors:** Cynthia C. Gilmour, Allyson L. Bullock, Alyssa McBurney, Mircea Podar, Dwayne A. Elias

**Affiliations:** a Smithsonian Environmental Research Center, Edgewater, Maryland, USA; b Biosciences Division, Oak Ridge National Laboratory, Oak Ridge, Tennessee, USA; University of Massachusetts Amherst

**Keywords:** *Archaea*, methylmercury, bioavailability, complexation, cysteine, *hgcAB*, mercury, methyltransferase, methylation, thiols

## Abstract

Methylmercury (MeHg) production was compared among nine cultured methanogenic archaea that contain *hgcAB*, a gene pair that codes for mercury (Hg) methylation. The methanogens tested produced MeHg at inherently different rates, even when normalized to growth rate and Hg availability. Eight of the nine tested were capable of MeHg production greater than that of spent- and uninoculated-medium controls during batch culture growth. Methanococcoides methylutens, an *hgcAB*^*+*^ strain with a fused gene pair, was unable to produce more MeHg than controls. Maximal conversion of Hg to MeHg through a full batch culture growth cycle for each species (except M. methylutens) ranged from 2 to >50% of the added Hg(II) or between 0.2 and 17 pmol of MeHg/mg of protein. Three of the species produced >10% MeHg. The ability to produce MeHg was confirmed in several *hgcAB*^*+*^ methanogens that had not previously been tested (Methanocella paludicola SANAE, Methanocorpusculum bavaricum, Methanofollis liminatans GKZPZ, and Methanosphaerula palustris E1-9c). Maximal methylation was observed at low sulfide concentrations (<100 μM) and in the presence of 0.5 to 5 mM cysteine. For M. hollandica, the addition of up to 5 mM cysteine enhanced MeHg production and cell growth in a concentration-dependent manner. As observed for bacterial Hg methylators, sulfide inhibited MeHg production. An initial evaluation of sulfide and thiol impacts on bioavailability showed methanogens responding to Hg complexation in the same way as do *Deltaproteobacteria*. The mercury methylation rates of several methanogens rival those of the better-studied Hg-methylating sulfate- and iron-reducing *Deltaproteobacteria*.

## IMPORTANCE

Archaea, specifically methanogenic organisms, play a role in mercury methylation in nature, but their global importance to MeHg production and the subsequent risk to ecosystems are not known. Methanogenesis has been linked to Hg methylation in several natural habitats where methylmercury production incurs risk to people and ecosystems, including rice paddies and permafrost. In this study, we confirm that most methanogens carrying the *hgcAB* gene pair are capable of Hg methylation. We found that methylation rates vary inherently among *hgcAB*^*+*^ methanogens but that several species are capable of MeHg production at rates that rival those of the better-know Hg-methylating sulfate- and iron-reducing bacteria. Methanogens may need to be considered equally with sulfate and iron reducers in evaluations of MeHg production in nature.

## INTRODUCTION

The discovery of the *hgcAB* gene pair led to the identification of several groups of microorganisms not previously identified as capable of mercury methylation, including methanogens. With the exception of a few syntrophic and fermentative *Firmicutes* (4), demonstrated mercury (Hg) methylators are all strictly anaerobic bacteria that use sulfate, iron, and carbon dioxide as terminal electron acceptors. Several *hgcAB*^*+*^ putative methylators have been identified in the phyla *Bacteroidetes*, *Chloroflexi*, and *Nitrospirae* (2) and other phyla, but none of these have been confirmed to produce methylmercury (MeHg) in culture. Overall, ~210 microbial species containing *hgcAB* have been identified among all of the bacteria and Archaea whose genomes have been sequenced, numbering >10,000.

The first list of *hgcAB*^*+*^ organisms was published in 2013 (3), with subsequent updates (2, 4). In all cases, the list has been based on *in silico* screening for both *hgcA* and *hgcB* by using the encoded amino acids to eliminate genomic variability such as codon wobble and species-specific nucleotide bias. Screening for *hgcA* included the presence of the distinctive conserved motif NVWCAAGK in the active methyltransferase site and predicted C-terminal transmembrane helices. The ferredoxin-encoding gene *hgcB* is generally, but not always, immediately downstream from *hgcA*. All but two of the *hgcAB*^*+*^ methanogens identified are in one class, *Methanomicrobia*. However, the overall importance of methanogens to MeHg production in nature is not well understood. Hg methylation has been confirmed in culture for only four organisms to date, Methanospirillum hungatei (5), Methanomethylovorans hollandica, Methanolobus tindarius (4), and Methanomassiliicoccus luminyensis (2). Here, we examined the Hg methylation rates of several strains to understand how their MeHg production compares to that of the better-studied methylators in the *Deltaproteobacteria*.

It is clear that there are several habitats, like rice paddies (1), some freshwater sediments (6), and periphyton (7, 8), where methanogens play an important role in MeHg production. However, their global importance to MeHg production and the subsequent risk to ecosystems are not known. A survey of publically available metagenomes showed that methanogens may also be dominant Hg methylators in termite hindguts, permafrost peat, Arctic sediments, and some bioreactors (2). Early work with clade-specific primers for *hgcAB*^*+*^ methanogens, *Deltaproteobacteria*, and *Firmicutes* suggested that archaeal Hg methylators are important in freshwater sediments from East Fork Poplar Creek, TN (9, 10).

In this study, several cultured representatives of *hgcAB*^*+*^ methanogens were used to understand the extent to which these organisms can produce MeHg and to begin to evaluate the biogeochemical controls on Hg methylation by methanogens. Specifically, we asked whether *hgcAB*^*+*^ methanogens have the ability to methylate significant portions of the available Hg, how methylation rates vary among species and metabolisms, and how the methylation rates of methanogens compare to those of *Deltaproteobacteria* and *Firmicutes*. We examined how the ability to methylate Hg is clustered within the Archaea, phylogenetically and metabolically.

Last, we began to evaluate the Hg complexes that are available for uptake and methylation by *hgcAB*^*+*^ methanogens. We wanted to know if the same types of Hg complexes that are available to the best-known group of Hg methylators, sulfate- and iron-reducing *Deltaproteobacteria*, are also available to Hg-methylating methanogens. Small thiols appear to be most bioavailable for methylation by sulfate-reducing bacteria (SRB) and iron-reducing bacteria (FeRB) (11, 12). Sulfide reduces the bioavailability of Hg to Hg-methylating *Deltaproteobacteria* via precipitation of mercuric sulfides that are less available for uptake (13–15). Dissolved organic matter (DOM) can slow the precipitation of nanoparticulate HgS (HgS_np_) (16–18), enhancing bioavailability to SRB (13, 14). The characteristics of DOM, including its aromaticity (19), thiol content, and degree of sulfidization (20), all influence how DOM impacts Hg and HgS_np_ bioavailability to SRB. Understanding the range of methylation rates and the biogeochemical controls on Hg methylation by methanogens is a critical part of understanding the role of methanogens in MeHg production and risk in nature.

## RESULTS

### Methylation results.

We quantified and compared the MeHg production of 9 of the now 19 identified *hgcAB*^+^ methanogens, all *Methanomicrobia* (see Table S1 in the supplemental material). The species tested included previously untested *hgcAB*^+^ methanogens (Methanocella paludicola SANAE, Methanocorpusculum bavaricum, Methanofollis liminatans GKZPZ, Methanococcoides methylutens, and Methanosphaerula palustris E1-9c) plus previously reported methylators Methanospirillum hungatei JF-1 (5), Methanomethylovorans hollandica, Methanolobus tindarius (4). Additionally, the bioavailability of Hg sulfide and Hg thiol complexes to methanogens was tested for selected species. Full information is provided in Data Set S1.

10.1128/mBio.02403-17.1 DATA SET S1  Data on the cultures and controls used in this study. Download DATA SET S1, XLSX file, 0.03 MB. Copyright © 2018 Gilmour et al. 2018 Gilmour et al. This content is distributed under the terms of the Creative Commons Attribution 4.0 International license.

10.1128/mBio.02403-17.3 TABLE S1 Methanogen strains used in this study. Download TABLE S1, PDF file, 0.04 MB. Copyright © 2018 Gilmour et al. 2018 Gilmour et al. This content is distributed under the terms of the Creative Commons Attribution 4.0 International license.

**FIG 1:**
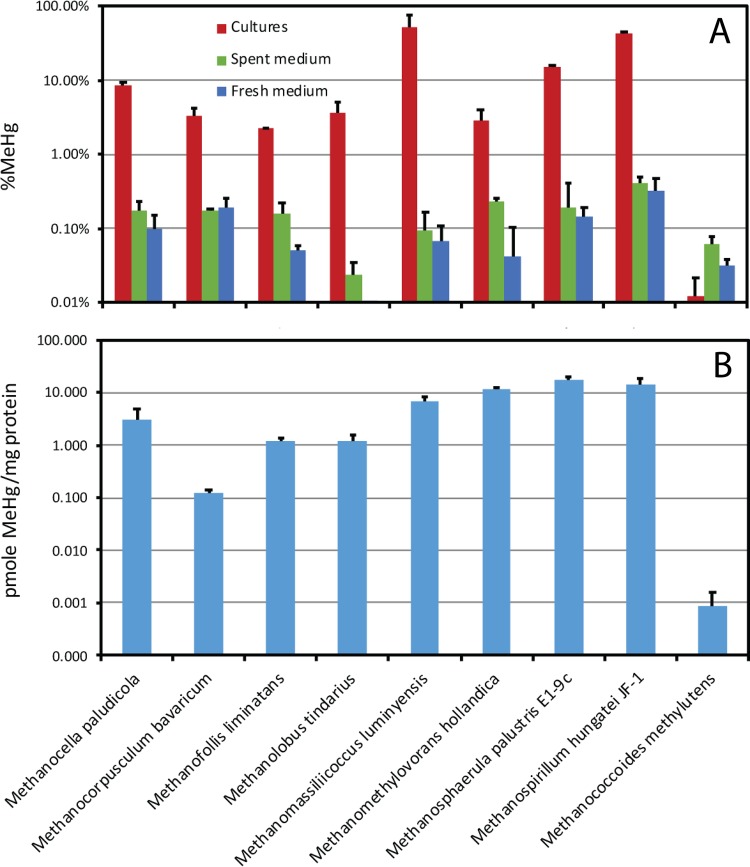
Maximal conversion of inorganic Hg to MeHg during batch culture growth by each *hgcAB*^+^ methanogen tested, expressed two ways. (Top) MeHg as a percentage of the total measured Hg in the culture medium (both unfiltered) at the end of log growth. Green and blue bars show noncellular control values. (Bottom) MeHg normalized to protein measured at the end of the log growth phase. Note the log scales. Bars are averages of triplicate separate cultures or controls. Methylation was measured from a 1 nM ^201^Hg(II) spike. Error bars are based on the standard deviation of MeHg only. M. luminyensis data are from reference 2.

Figure 1 (top) provides a comparison of the maximum measured MeHg production levels of all nine of the *hgcAB*^*+*^ methanogens tested. Methylation was assayed in batch cultures. MeHg concentrations were measured once cultures entered stationary phase, and percentage of MeHg formation was calculated on the basis of the total measured Hg concentration in the culture medium at the same time. Controls included MeHg production in fresh and spent media for each strain. Several organisms were tested with different concentrations of sulfide or cysteine in culture medium; the data in Fig. 1 are methylation rates under the most favorable observed conditions, usually between 0.5 and 4 mM cysteine and 10 and 100 μM sulfide.

Eight of the nine species tested were capable of more MeHg production than controls during batch culture growth. Maximal conversion of Hg to MeHg for each strain through a full batch culture growth cycle ranged from 2 to ~65%. Three of the species produced >10% MeHg, M. luminyensis, M. palustris, and M. hungatei. Using lower sulfide concentrations, M. tindarius and M. hollandica produced somewhat higher percentages of MeHg than previously reported. MeHg production by M. hungatei (43%) was similar to that reported by Yu et al. (5). Methanococcoides methylutens, a species with a fused *hgcAB* gene pair, did not produce more MeHg than controls in triplicate assays repeated three times.

There was significant loss of Hg from the aqueous phase in many of the methylation assays. Hg losses were comparable in controls (spent and fresh media) and cultures (see Data Set S1). Differences in Hg loss appear to be related to medium chemistry, with greater losses often observed in higher-sulfide media. However, no systematic tests of medium chemistry effects on Hg losses was done, nor were the mechanisms of loss (bottle sorption versus reduction to Hg°) systematically evaluated.

Although the percentage of MeHg is an easily understandable way to evaluate the magnitude of MeHg production by cultures, a better way to report and compare rates among organisms and conditions is methylation normalized to growth (Fig. 1, bottom). Protein-normalized MeHg production by the Hg-methylating methanogens ranged from roughly 1 to 20 pmol of MeHg/mg of protein, similar to that in prior assays of M. tindarius and M. hollandica. Yu et al. (5) reported about 100 pmol of MeHg/mg of protein for M. hungatei during log-phase growth, which was reduced to <10 pmol of MeHg/mg of protein in the presence of a sulfide reductant.

Protein-normalized MeHg production by *Deltaproteobacteria* and *Firmicutes*, measured by the same batch culture approach, ranged from about 1 to 600 and 1 to 50 pmol of MeHg/mg of protein, respectively, with Geobacter bemidjiensis and *Desulfovibrio* sp. strain ND132 the highest producers to date (4). A survey of eight *hgcAB*^*+*^ *Desulfovibrio* species produced between about 20 and 250 pmol of MeHg/mg of protein in short-term (3 h) washed cell assays with 500 μM cysteine and no added sulfide (21).

### Methylation by methanogenic Archaea in comparison to *Bacteria*.

**FIG 2:**
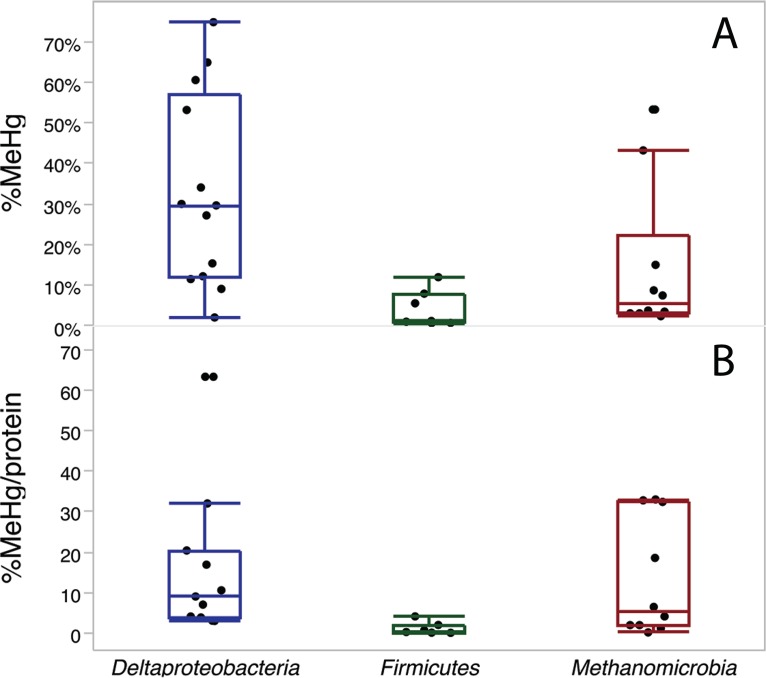
Comparison of maximal MeHg production among the three major clades of Hg-methylating bacteria. (Top) Maximal conversion of inorganic Hg to MeHg by 13 *Deltaproteobacteria*, 7 *Firmicutes*, and 10 *Methanomicrobia* species. (Bottom) MeHg production normalized to both the measured Hg and protein concentrations at the end of methylation assays, for the same organisms and assays, as (MeHg/Hg)/(mg of protein/liter). Hg spike levels ranged from 1 to 50 nM. Data combine assays done over the course of batch culture growth in this study, in the study described in reference 4, and during 3-h washed-cell assays (21). In these bar-and-whisker plots, the average value for each species is represented by a dot; the centerline, top, and bottom of each bar are the median and upper and lower quartiles, respectively, across all species; and the whiskers are drawn to the furthest data point within 1.5× the quartile value (thus excluding outliers).

To compare methylation abilities among clades and across studies with different growth conditions and Hg levels, we compared absolute conversion of Hg to MeHg (percentage of MeHg; Fig. 2, top) and also normalized MeHg production for each species to both protein and Hg (Fig. 2, bottom). In a past study, we found that methylation rates in washed-cell assays were proportional to the inorganic Hg(II) concentration (21). For this comparison, we chose the highest measured methylation rate across all of the conditions tested for each species measured, usually between 0.5 and 4 mM cysteine and 10 and 100 μM sulfide. Figure 2 compiles data from this study, our prior batch culture assays of all three clades (4), and washed-cell assays of *Desulfovibrio* species (21). We did not correct the methylation rates for time, although batch cultures were often incubated for several days while washed cell assays ran 3 h. Our observation has been that most MeHg production in cultures occurs in the first hours of incubation with active cells (12, 21). Nevertheless, the differences in timing, culture conditions, and Hg concentrations among assays add uncertainty to this comparison.

On average, across all of the species tested, *Deltaproteobacteria* produced the highest average percentage of MeHg and the highest percentage of MeHg normalized to protein. There are few *hgcAB*^*+*^ *Deltaproteobacteria* that convert <10% of Hg(II) to MeHg under ideal conditions (moderate thiol levels, low sulfide levels) during a few hours of log-phase growth. Of 13 species tested, 4 are able to convert more than half of the available Hg(II) to MeHg. However, normalized to protein and Hg, MeHg production by methanogens rivals that of *Deltaproteobacteria*. While methanogens tended to convert a smaller fraction of Hg(II) to MeHg during batch culture growth, their cell densities and growth rates in culture are usually lower than those of sulfate and iron reducers. Expressed as the percentage of MeHg per milligram of protein, several methanogens exhibited methylation rates equal to those of the best *Deltaproteobacteria*. Including this study, the methylation rates of about the same number of Deltaproteobacteria (13) and Methanomicrobia (9) have been tested quantitatively in culture. SRB and FeRB have been tested in several labs (4, 12, 15, 21–37), but only one *hgcAB*^*+*^ methanogen has been tested in culture outside our group (5).

About 40 *hgcAB*^*+*^ *Firmicutes* have been identified to date, but only 7 have been tested for methylation in culture so far (4). Almost all produced <10% MeHg in culture and a uniformly low percentage of MeHg per milligram of protein (Fig. 2). The majority of these are sulfite and sulfate reducers.

### Influence of cysteine on methylation by methanogens.

The presence of small thiols often enhances the bioavailability of Hg for methylation to *Deltaproteobacteria*. We evaluated Hg methylation by M. hollandica and M. tindarius across cysteine gradients to begin to assess the bioavailability of Hg thiols to *hgcAB*^*+*^ methanogens. Methanogens often require a source of reduced S for growth. Addition of cysteine, other thiols, or sulfide can stimulate growth, complicating the interpretation of Hg methylation data. In this study we used methionine, which is not a strong ligand for Hg, to provide reduced sulfur for growth. The amino acid methionine contains S in a thioether side chain but not a free thiol. By providing an alternative reduced-S source, we hoped to test the influence of cysteine on Hg bioavailability, separately from its influence on cell growth.

**FIG 3:**
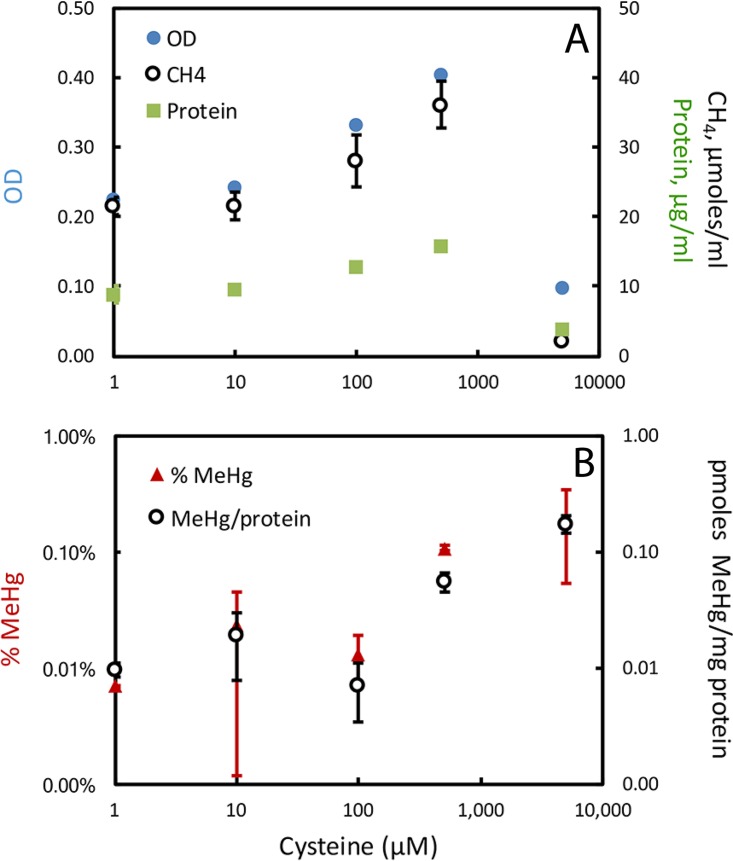
Impact of cysteine on M. hollandica growth and MeHg production. (Top) Growth assessed by OD and methane production. (Bottom) MeHg as a percentage of the total Hg in the culture medium and MeHg normalized to protein. All measurements were made once all cultures reached stationary phase (312 h). Methionine at 1 mM was included in all culture media. Sulfide was added to all at 10 μM, but the concentration was to 2 to 4 μM at the end of log-phase growth. Note the log scales. Error bars are standard deviations of triplicate cultures.

Cysteine enhanced the production of MeHg by both cultures. For M. hollandica, MeHg production was tested in batch culture across a cysteine gradient of 1 to 5,000 μM, with 1 mM methionine added to the medium. However, even when methionine was provided, cysteine up to 500 μM enhanced cell growth in a concentration-dependent manner (Fig. 3). Growth was inhibited at 5 mM cysteine. Methionine (1 mM) as the primary reduced-S source produced less growth than 500 μM cysteine (in comparison with a prior assay [4]). In prior experiments, M. hollandica grown in batch culture methylation assays with 500 μM cysteine, 50 μM sulfide, and 10 nM Hg produced ~1% MeHg and ~5 pmol of MeHg/mg of protein. Under those conditions, cultures grew well, achieving an optical density (OD) of ~0.5 and ~22 μg of protein/ml. Cysteine may be a more available form of reduced sulfur than methionine and also serves as an additional reductant (although all media were reduced with TiNTA (titanium nitrilotriacetic acid) and monitored visually with resazurin). Corrected for its effects on growth, cysteine enhanced the bioavailability of Hg for methylation by M. hollandica. The absolute amount of MeHg produced increased with the cysteine concentration in the medium, as did MeHg production normalized to cell protein, with the most MeHg production in the 5 mM cysteine cultures, despite their relatively weak growth.

**FIG 4:**
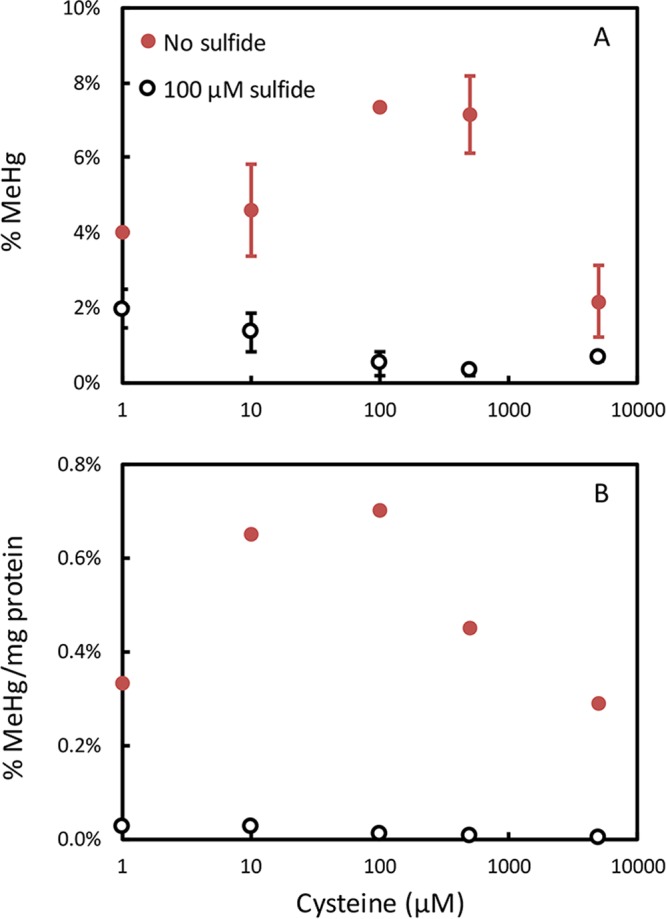
Impact of cysteine on MeHg production by M. tindarius in medium without (red) and with (black) 100 μM sulfide and 500 μM methionine. (Top) MeHg as a percentage of the total Hg in the culture medium. (Bottom) MeHg production normalized to both the measured Hg and protein concentrations at the end of methylation assays, as (MeHg/Hg)/(mg of protein/liter). All measurements were made once all cultures reached stationary phase. Error bars are standard deviations of triplicate cultures. Growth data are in Fig. S1.

Cysteine also enhanced methylation by M. tindarius. M. tindarius was grown across the same cysteine gradient in medium with or without the addition of 100 μM sulfide and 500 μM methionine (Fig. 4). Cysteine enhanced growth more strongly in the medium without added sulfide or methionine. In medium without added sulfide, MeHg as a fraction of the Hg in the medium (at the end of incubation) and MeHg production normalized to cell protein both increased up to about 100 μM cysteine but declined as the cysteine concentration increased further. Sulfide had a strong inhibitory effect on methylation. MeHg production was lower in medium with added sulfide, and the addition of cysteine did not increase MeHg production, even though growth was much stronger. The sulfide concentration in cultures without added sulfide averaged 0.3 μM at the end of log-phase growth; cultures with 100 μM added sulfide averaged 1.4 μM. In medium with or without sulfide, cysteine held Hg in solution in the culture medium in a concentration-dependent way, based on measurement of the Hg remaining in the aqueous medium (including cells) at the end of growth (Fig. S1). Interestingly, in the absence of added sulfide, cysteine increased methylation even when normalized to the final Hg concentration, suggesting that the mechanism for cysteine enhancement is not just its ability to enhance Hg solubility, but that the Hg-cysteine complex is more available than other forms. This is consistent with findings on *Deltaproteobacteria* (11, 21).

10.1128/mBio.02403-17.2 FIG S1  Impact of cysteine on M. tindarius growth and MeHg production. Download FIG S1, PDF file, 0.05 MB. Copyright © 2018 Gilmour et al. 2018 Gilmour et al. This content is distributed under the terms of the Creative Commons Attribution 4.0 International license.

### Influence of sulfide on Hg methylation by methanogens.

**FIG 5:**
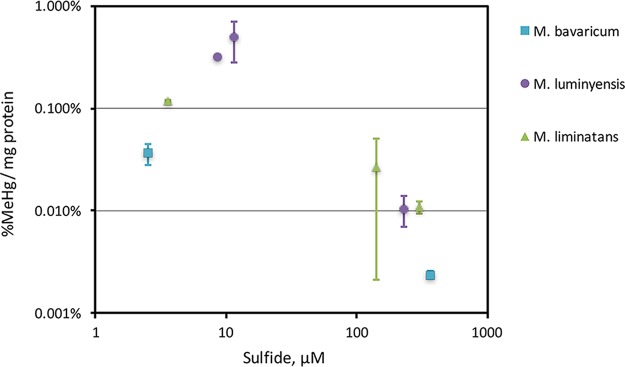
Impact of sulfide on MeHg production by three methanogens based on values measured at the end of log growth in triplicate batch cultures. Sulfide was added to all cultures (between 10 and 1,000 μM); the sulfide concentration shown was measured at the end of the log phase of growth. MeHg production is normalized to both the measured Hg and protein concentrations at the end of methylation assays, as (MeHg/Hg)/(mg of protein/liter). All cultures were grown with 4 mM cysteine. Error bars are standard deviations of triplicate cultures. All measurements were made once all cultures reached stationary phase.

Sulfide reduces the bioavailability of Hg to Hg-methylating *Deltaproteobacteria* via precipitation of mercuric sulfides that are less available for uptake (13–15). Sulfide also inhibited MeHg production by three *hgcAB*^*+*^ methanogens grown in medium with sulfide additions between 10 and 1,000 μM (Fig. 5). Sulfide additions resulted in loss of Hg from medium to either bottle walls or reduction (see Fig. S1 for an example). Even so, sulfide reduced MeHg production normalized to the final measured Hg concentration in the culture medium.

## DISCUSSION

### Newly identified Hg-methylating methanogens.

This is the first confirmation of methylation ability in culture of *hgcAB*^*+*^ Methanocella paludicola SANAE, Methanocorpusculum bavaricum, Methanofollis liminatans GKZPZ, and Methanosphaerula palustris E1-9c. Methanocella paludicola SANAE is the first confirmed methylator in the order *Methanocellales*. We identified a second organism with a fused *hgcAB* gene pair that is unable to produce MeHg under the conditions tested, Methanococcoides methylutens; the first was Pyrococcus furiosus (2).

### Rarity of *hgcAB* among methanogens.

**FIG 6:**
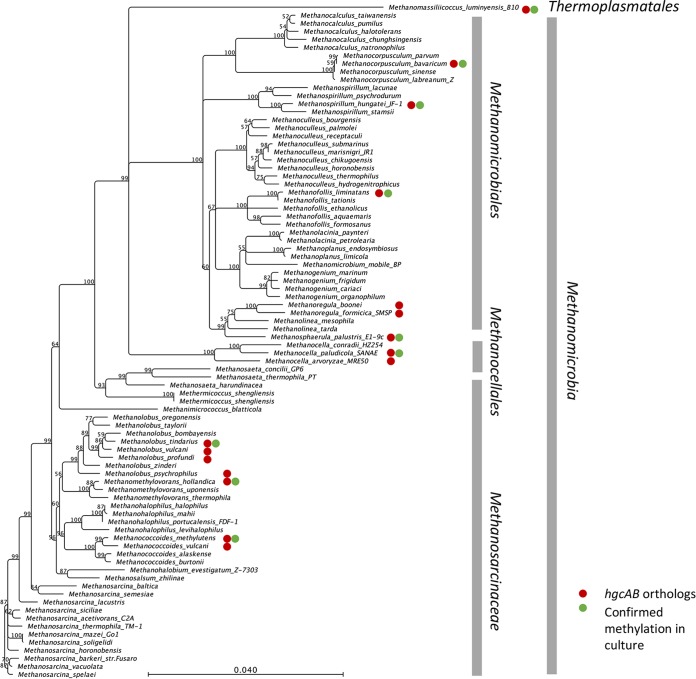
16S rRNA gene distance phylogeny of the *Methanomicrobia/Thermoplasmata* superclass (excluding the Halobacteria) within the methanogenic *Euryarchaeota*, showing all of the available sequenced genomes. Red circles show organisms with *hgcAB* orthologs. Green circles are organisms confirmed to methylate Hg in culture. Branch points supported with bootstrap values of ≥50% are shown. The scale bar indicates 0.04 substitution per nucleotide position.

To date, *hgcAB* orthologs have been identified in only 18 sequenced *Euryarchaeota* genomes, among several dozen sequenced genomes (Fig. 6). Methylation in culture has been confirmed for eight of those. All but two of the identified Hg methylators are in one class, *Methanomicrobia* (*Methanomassiliicoccus* has tentatively been placed in the class Thermoplasmata [38]). There are no identified *hgcAB*^*+*^ organisms in the other four classes of methanogens, including the Methanobacteria/Methanococci superclass, Methanopyri, or the newly identified WSA2 class (“*Candidatus* Methanofastidiosa”) (39). Among the members of the class *Methanomicrobia*, most of the *hgcAB*^*+*^ strains are in the two larger orders *Methanomicrobiales* and *Methanosarcinaceae*. Hg methylators are most common in the genera *Methanolobus*, *Methanoregula*, and *Methanocella* but, aside from these small clusters, are scattered throughout a 16S rRNA gene-based phylogeny. In the two methanogenic orders that contain *hgcAB*^*+*^ organisms, *Methanomicrobia* and Thermoplasmata, there are currently about 85 sequenced genomes. About 20% of those species are *hgcAB*^*+*^.

In comparison, *hgcAB* orthologs are found in 5 of the 10 orders of *Deltaproteobacteria* (*Desulfarculales*, *Desulfobacterales*, *Desulfovibrionales*, *Desulfuromonadales*, and *Syntrophobacterales*). Overall, there are ~110 *hgcAB*^*+*^ species among the >1,000 identified species in these five orders, or roughly 10% of the species identified. Known Hg methylators are concentrated in the genus *Desulfovibrio* in the order *Desulfovibrionales* (26 species, or about 25% of the named species) and in the genus *Geobacter* in the order *Desulfuromonadales* (19 species). Almost all of the members of the genus *Geobacter* whose genomes have been sequenced contain *hgcAB*. In the large order *Desulfobacterales*, there are 22 identified species with *hgcAB* orthologs (Hg methylation has been demonstrated in 7 of them) spread among several genera and >250 identified species.

### Carbon metabolism of Hg-methylating methanogens.

Substrates for methanogenesis include H_2_-CO_2_, acetate, and methylated compounds. Among the methanogens whose genomes have been sequenced, Hg methylators were found among the methylotrophic and hydrogenotrophic methanogens but not among the small group of described aceticlastic methanogens. None of the hydrogenotrophic Hg methylators are class I methanogens (40). This distribution was also observed in a broad query of available microbial metagenomes (2).

Several methylotrophic *hgcAB*^*+*^ methanogens were identified in the order *Methanosarcinales*, the only methanogens that contain cytochromes and can metabolize methylated compounds via a membrane-bound electron transport chain. Most of the Hg-methylating members of the order *Methanosarcinales* are *Methanolobus* species, which are all methylotrophic and somewhat salt tolerant. Substrates for some of the species include methanol (MeOH), methylamines, and methyl sulfides. We tested Hg methylation by Methanolobus tindarius growing on MeOH under a N_2_-CO_2_ atmosphere. The other *hgcAB*^*+*^ *Methanosarcinales* member is M. hollandica, which is also obligately methylotrophic. We tested its methylation ability on trimethylamine-MeOH under a N_2_-CO_2_ atmosphere.

Other methylotrophic methanogens are obligately H_2_ dependent. *Methanomassiliicoccus* is a recently identified genus of obligately methylotrophic and H_2_-dependent methanogens isolated from the intestinal tracts of humans, ruminants, and termites; anaerobic digesters; and soils. Along with *Methanoplasma*, *Methanogranum*, and *Methanomethylophilus*, these organisms have been classified as a new, seventh order of methanogens, *Methanomassiliicoccales*, in the class *Thermoplasmatales*, phylogenetically distant from other methanogenic orders (41). The order lacks the pathway for CO_2_ reduction to methyl coenzyme M and produces methane by H_2_-dependent reduction of MeOH or methylamines. The two known species of *Methanomassiliicoccus* both contain *hgcAB*. Methanomassiliicoccus luminyensis B10 produced MeHg in cultures growing on MeOH supplemented with rumen fluid (2).

The remaining *hgcAB*^*+*^ methanogens are obligately hydrogenotrophic organisms in the orders *Methanomicrobiales* and *Methanocellales*. The order *Methanomicrobiales* is a highly polyphyletic group of hydrogenotrophic methanogens, but with a narrow substrate range, generally utilizing only CO_2_, with either formate or H_2_ as the reducing agent. We identified eight species with *hgcAB* orthologs in four families. Most were in the family *Methanoregulaceae*, formerly the E1/E2 and R10 groups or “fen cluster” phylotypes (42). Five *Methanoregulaceae* species in three genera, *Methanoregula*, *Methanosphaerula*, and *Methanolinea*, have been sequenced (42). Neither of the two *Methanolinea* strains contain *hgcAB*, but both *Methanoregula* species (M. boonei and M. formicica) and the single *Methanosphaerula* species (M. palustris) do. Both M. boonei and M. palustris were isolated from oligotrophic peat environments (43, 44). M. formicica was isolated from an anaerobic sludge reactor treating brewery effluent (45). The unclassified *Methanoregulaceae* archaeon JGI M3C4D3-001-G22, whose genome has recently been sequenced, is also *hgcAB*^*+*^. Hg methylation was tested in Methanosphaerula palustris E1-9c cultures growing in H_2_-CO_2_ supplemented with acetate.

Two other methylators in the order *Methanomicrobiales* were identified. Methanospirillum hungatei is the only organism in the family *Methanospirillaceae* whose genome has been sequenced. Various phylogenies place M. hungatei within or just outside *Methanoregula* (46). Isolated from sewage sludge, it was one of the earliest methanogens characterized (47) and the first confirmed Hg-methylating methanogen (5). We grew this organism in H_2_-CO_2_ supplemented with acetate and formate for Hg methylation testing. Methanocorpusculum bavaricum (family *Methanocorpusculaceae*) was isolated from a sugar wastewater pond. In addition to H_2_-CO_2_ methanogenesis, it can utilize secondary alcohols as electron donors to reduce CO_2_ and requires rumen fluid in culture. To test Hg methylation, this organism was cultured under H_2_-CO_2_ in medium supplemented with acetate, formate, rumen fluid, and fatty acids.

To summarize, the *hgcAB* gene pair appears to be confined to two classes of methanogens, *Methanomicrobia* and Thermoplasmata, but is found in organisms with a wide variety of pathways for methanogenesis. Methanosarcina may metabolize one-carbon compounds like MeOH, acetate, and methylamines via a membrane-bound electron transport chain. Other methylotrophic methanogens containing *hgcAB* require H_2_. Our methylation assays of several previously untested *hgcAB*^*+*^ *Methanomicrobia* species lead us to believe that these methanogens should be considered equally with sulfate and iron reducers in evaluations of potential Hg-methylating biogeochemical conditions in nature. Our understanding of the distribution and activity of *hgcAB*^*+*^ organisms in nature and of the controls on *hgcAB* expression remains in its infancy.

## MATERIALS AND METHODS

### Methanogen strains evaluated.

The methanogen strains used in this study are listed in Table S1 along with the sources of the cultures and the growth media used. Species purity was checked through 16S rRNA gene nucleotide sequencing.

### Hg methylation assays.

MeHg production was evaluated by measuring the production of Me^201^Hg from a 1 or 10 nM inorganic >98% enriched ^201^Hg spike during triplicate batch culture growth (4). The culture media and growth conditions used for methylation assays are described in Table S2. Several cultures were tested under a range of added thiol and sulfide levels. Controls included fresh and spent culture media (filtrate of mature cultures) under matched redox conditions. Spent medium was prepared by anaerobic filtration of late-log- or stationary-phase cultures. Organisms without *hgcAB* were used as negative-control strains. All assays and controls were conducted in separate, triplicate bottles. Preparation of medium and spent-medium controls was done under O_2_-free N_2_ or other reduced headspace gases, as appropriate for each culture. Several cultures were tested multiple times (Data Set S1).

10.1128/mBio.02403-17.4 TABLE S2 Culture media and growth conditions used in this study. Download TABLE S2, PDF file, 0.1 MB. Copyright © 2018 Gilmour et al. 2018 Gilmour et al. This content is distributed under the terms of the Creative Commons Attribution 4.0 International license.

During batch culture growth and Hg methylation assays, cell density, protein concentration, sulfide, and headspace methane were measured to assess growth and monitor redox and medium chemistry (4). Some culture media contained added sulfide; in other media, cells produced sulfide from added cysteine. Culture media were amended with additional cysteine, methionine, or sulfide as noted for methylation assays. For most assays, medium recipes were amended with 500 μM cysteine. The Hg-Cys complex is highly bioavailable to Hg-methylating *Deltaproteobacteria* (11, 12, 21).

Methylation was calculated as the concentration of excess Me^201^Hg in unfiltered culture medium at the end of log-phase growth divided by the measured concentration of excess total ^201^Hg measured in the same sample. This provides a maximal estimate of methylation, given the loss of Hg(II) from culture medium to bottle walls and potentially to reduction during growth. Cultures were considered to be methylators when they produced significantly more MeHg than matched fresh- and spent-medium controls. Since small amounts of MeHg can be produced chemically (48), it is important to assess MeHg production in appropriate controls.

### Hg and MeHg analysis.

All measurements were made by isotope dilution inductively coupled plasma mass spectrometry (ICP-MS) as described previously (4). Briefly, the total Hg in filtered or unfiltered samples was measured after digestion, SnCl_2_ reduction, purging, and trapping. MeHg was determined following aqueous phase distillation and ethylation. Me^199^Hg isotope dilution standards were synthesized in house from ^199^HgCl_2_ (Oak Ridge National Laboratory; 91.95% enriched) (49). All measurements were made with Brooks Rand MERX automated systems (Brooks Rand Instruments, Seattle, WA) interfaced with a PerkinElmer Elan DRC II ICP-MS apparatus (PerkinElmer Inc., Shelton, CT). The detection limit for Me^201^Hg and total ^201^Hg averaged 0.9 and 27 pM, respectively, in the dilutions used for analysis. The average MeHg percentage in controls was 0.7% of the aqueous ^201^Hg.

### 16S rRNA gene phylogeny.

A distance scaffold and supporting multiple-sequence alignment were constructed with CLC Sequence Viewer 7 (Qiagen) from 16S rRNA gene sequences obtained from GenBank and JGI. The phylogeny used a neighbor-joining algorithm with Jukes-Cantor nucleotide distance measurement and 1,000 bootstrap runs.

10.1128/mBio.02403-17.5 TABLE S3 Genome accession numbers, phylogeny, culture source, and references for original isolation of the methanogens used in this study. Download TABLE S3, PDF file, 0.1 MB. Copyright © 2018 Gilmour et al. 2018 Gilmour et al. This content is distributed under the terms of the Creative Commons Attribution 4.0 International license.
