# Supplementary material for: Robust Mercury Methylation across Diverse Methanogenic Archaea
Source: mBio. 2018 Apr 10;9(2):e02403-17. doi: 10.1128/mBio.02403-17 (PMC5893877; doi:10.1128/mBio.02403-17)
Supplement: TABLE S1 [file mbo001183828st1.pdf]

Table S1. Methanogen strains used in the study, including the source of the culture.

| Strain | Culture collection # | Source of culture |
|---|---|---|
| ***hgcAB+*** | | |
| *Methanocella paludicola* SANAE | DSM 17711 | Gift from Sanae Sakai |
| *Methanocorpusculum bavaricum* | DSM 4179 | DSMZ |
| *Methanofollis liminatans* GKZPZ | DSM 4140 | DSMZ |
| *Methanosphaerula palustris* E1-9c | DSM 19958 | Gift from Hinsby Cadillo-Quiroz |
| *Methanospirillum hungatei* JF-1 | DSM 864 | Gift from Hinsby Cadillo-Quiroz |
| *Methanolobus tindarius* | DSM 2278 | DSMZ |
| *Methanomethylovorans hollandica* | DSM 15978 | DSMZ |
| *Methanomassiliicoccus luminyensis* B10 | DSM 25720 | DSMZ |
| *Methanococcoides methylutens* * | DSM 2657 | Gift from Kevin Sowers |
| ***hgcAB-*** | | |
| *Methanoculleus bourgensis* MS2 | ATCC 43281 | ATCC |
| *Methanobrevibacter smithii* | ATCC 35061 | ATCC |

* *hgcAB* gene pair is fused
