# Supplementary material for: Robust Mercury Methylation across Diverse Methanogenic Archaea
Source: mBio. 2018 Apr 10;9(2):e02403-17. doi: 10.1128/mBio.02403-17 (PMC5893877; doi:10.1128/mBio.02403-17)
Supplement: FIG S1 [file mbo001183828sf1.pdf]

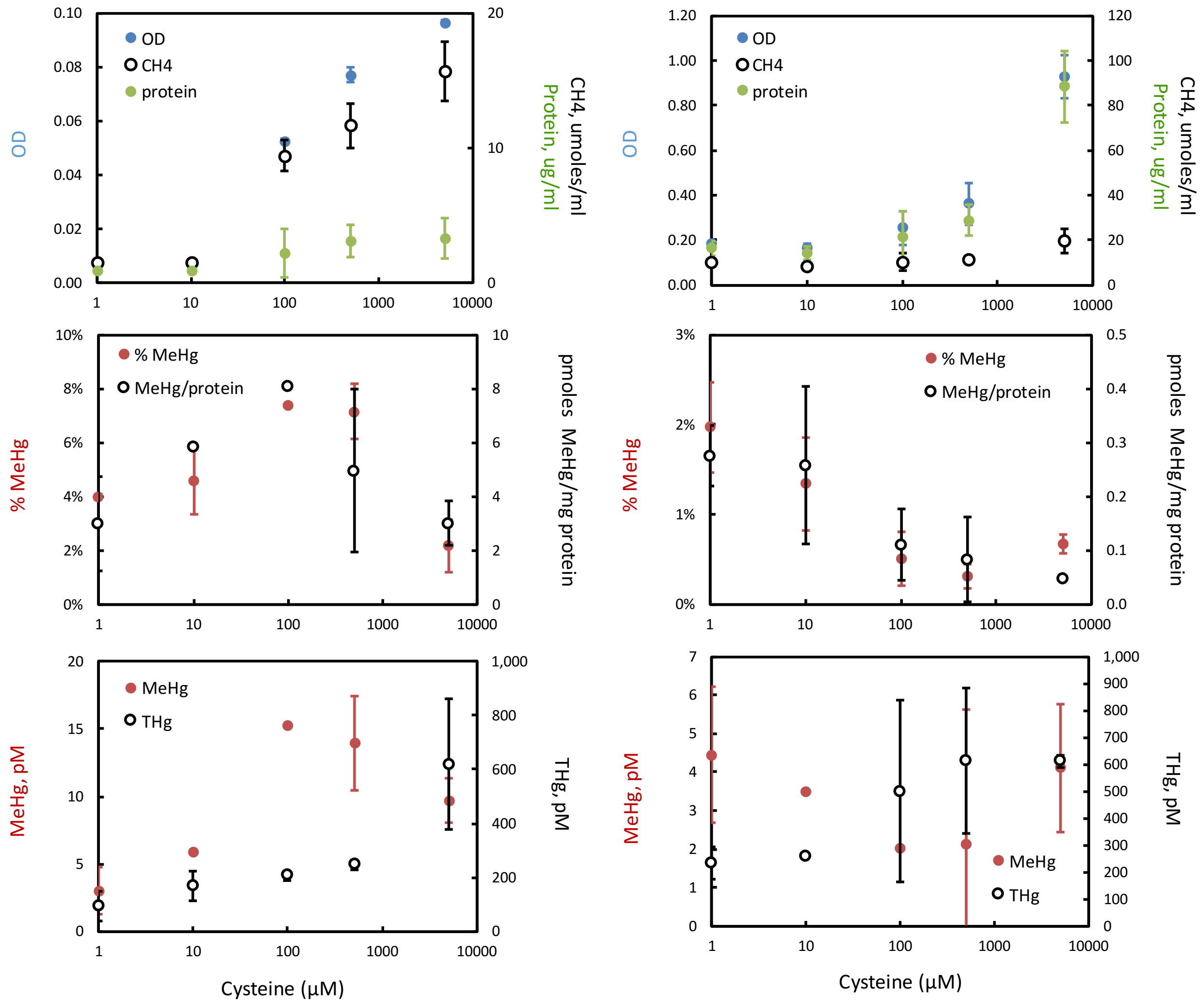

Figure S1. Impact of cysteine on growth and MeHg production by *M. tindarius*. Left, batch culture growth with no added sulfide or methionine; right growth in medium with 100 µM sulfide and 500 µM methionine. All media were supplemented with 1 nM ^{201}Hg. Growth medium is from Table S2. Top, growth assessed by optical density, protein and methane production. Middle, MeHg as a percentage of the total Hg in culture medium, and MeHg normalized to protein. Bottom, raw total and MeHg data. All measurements were made once all cultures reached stationary phase.
