# Supplementary material for: Robust Mercury Methylation across Diverse Methanogenic Archaea
Source: mBio. 2018 Apr 10;9(2):e02403-17. doi: 10.1128/mBio.02403-17 (PMC5893877; doi:10.1128/mBio.02403-17)
Supplement: TABLE S2 [file mbo001183828st2.pdf]

Table S2. Culture media and growth conditions for methanogens used in the study.

Strain	Substrate(s)	Growth Temp.	Headspace	Growth pH	Growth medium Reference
hgcAB+					
M. paludicola	acetate 1 mM	37	20 psi 80% H_2/20% CO_2	7.5	(1)
M. bavaricum	formate 30 mM, acetate 60 mM, rumen fluid, FA mix	37	20 psi 80% H_2/20% CO_2	7.5	(1)
M. liminatans	formate 30 mM, acetate 25 mM	37	20 psi 80% H_2/20% CO_2	7.2	(1)
M. palustris	acetate 4 mM	30	20 psi 80% H_2/20% CO_2	5.6	(1)
M. hungatei	formate 30 mM, acetate 5 mM	34	20 psi 80% H_2/20% CO_2	7.5	(1)
M. tindarius	MeOH 125 mM	25	15 psi 80% N_2/20% CO_2	6.5	(2)
M. hollandica	TMA 34 mM, MeOH 0.5%	37	15 psi 80% N_2/20% CO_2	6.5	(2)
M. luminyensis	Acetate 20 mM, Trypticase Peptone, MeOH, YE, rumen fluid, FA mix	37	20 psi 80% H_2/20% CO_2	7.5	(3)
M. methylutens *	40 mM TMA/25 mM MeOH	30	15 psi N2	7.2	Sowers, personal communication#
hgcAB-					
M. bourgensis	formate 44 mM, acetate 12 mM	37	20 psi 80% H_2/20% CO_2	7	(2)
M. smithii	formate 44 mM, acetate 12 mM	37	20 psi 80% H_2/20% CO_2	7	(2)

*Fused *hgcAB*

#*M. methylutens* was grown on DSM medium 280, as modified by Sowers (pers. comm). Substrates were trimethylamine (42 mM) and MeOH (24 mM). Medium salts were NaCl (400 mM); KCl (10 mM); NH_4Cl (10 mM), $MgSO_4$ (50 mM), $CaCl_2$ (1 mM) and $Fe(NH_4)_2(SO_4)_2$ (10 μM). The medium was buffered with 20 mM carbonate/bicarbonate and 25 mM phosphate to pH 7.2 Trace element and vitamin mixes from DSM medium 141 were both used a 10 ml/L. Sulfide was added to 100 μM and L-cysteine to 500 μM.

References:

1. Christensen GA, Wymore AM, King AJ, Podar M, Hurt RA, Santillan EU, Soren A, Brandt CC, Brown SD, Palumbo AV. 2016. Development and validation of broad-range qualitative and clade-specific quantitative molecular probes for assessing mercury methylation in the environment. Applied and Environmental Microbiology 82:6068-6078.
2. Gilmour C, Podar M, Bullock AL, Graham AM, Brown SD, Somenahally AC, Johs A, Hurt RA, Jr., Bailey KL, Elias DA. 2013. Mercury Methylation by Novel Microorganisms from New Environments. Environmental Science & Technology 47:11810-11820.
3. Podar M, Gilmour CC, Brandt CC, Soren A, Brown SD, Crable BR, Palumbo AV, Somenahally AC, Elias DA. 2015. Global prevalence and distribution of genes and microorganisms involved in mercury methylation. Science advances 1:e1500675.
