# Supplementary material for: Robust Mercury Methylation across Diverse Methanogenic Archaea
Source: mBio. 2018 Apr 10;9(2):e02403-17. doi: 10.1128/mBio.02403-17 (PMC5893877; doi:10.1128/mBio.02403-17)
Supplement: TABLE S3 [file mbo001183828st3.pdf]

Table S3. Genome accession numbers, phylogeny, culture source and references for original isolation for methanogens used in the study.

Strain	Genome Accession Number	Class	Order	Family	Source habitat	Location	Species Reference
			hgcAB+				
M. paludicola	AP011532.1	*Methanomicrobia*	*Methanocellales*	*Methanocellaceae*	rice paddy	Japan	(1)
M. bavaricum	AUMX01000002	*Methanomicrobia*	*Methanomicrobiales*	*Methanocorpusculaceae*	anaerobic sediment of wastewater treatment plant of a sugar factory	Suedzucker company, Regensburg, Germany	(2)
M. liminatans	CM001555.1	*Methanomicrobia*	*Methanomicrobiales*	*Methanomicrobiaceae*	industrial wastewater treatment plant	Germany	(3)
M. palustris	CP001338.1	*Methanomicrobia*	*Methanomicrobiales*	*E1/E2*	peatlands, minerotrophic fen	New York	(4)
M. hungatei	CP000254.1	*Methanomicrobia*	*Methanomicrobiales*	*Methanospirillaceae*	sewage sludge	US	(5)
M. tindarius	AZAJ01000001.1	*Methanomicrobia*	*Methanosarcinales*	*Methanosarcinaceae*	lake sediment	Lakes of Marinello, Italy	(6)
M. hollandica	CP003362.1	*Methanomicrobia*	*Methanosarcinales*	*Methanosarcinaceae*	anaerobic sediment eutrophic freshwater pond	Netherlands	(7)
M. luminyensis	NZ_CAJE00000000.1	*Thermoplasmata*	*Thermoplasmata*	*Methanomassiliicoccus*	human feces	Marseille, France	(8)
M. methylutens	NZ_JRHO00000000	*Methanomicrobia*	*Methanosarcinales*	*Methanosarcinaceae*	marine sediment, submarine canyon	Off the coast of southern CA	(9)
			hgcAB-				
M. bourgensis	HE964772.2	*Methanomicrobia*	*Methanomicrobiales*	*Methanomicrobiaceae*	tannery by-products/sewage sludge digester	France	(10)
M. smithii	CP000678.1	*Methanobacteria*	*Methanobacteriales*	*Methanobacteriaceae*	human feces	US	(11)

References:

1. Sakai S, Imachi H, Hanada S, Ohashi A, Harada H, Kamagata Y. 2008. *Methanocella paludicola* gen. nov., sp nov., a methane-producing archaeon, the first isolate of the lineage 'Rice Cluster I', and proposal of the new archaeal order *Methanocellales* ord. nov. International Journal of Systematic and Evolutionary Microbiology 58:929-936.
2. Zellner G, Stackebrandt E, Messner P, Tindall B, de Macario EC, Kneifel H, Sleytr U, Winter J. 1989. *Methanocorpusculaceae* fam. nov., represented by *Methanocorpusculum parvum*, *Methanocorpusculum sinense* spec. nov. and *Methanocorpusculum bavaricum* spec. nov. Archives of microbiology 151:381-390.
3. Zellner G, Sleytr UB, Messner P, Kneifel H, Winter J. 1990. *Methanogenium-liminatans* spec-nov, a new coccoid, mesophilic methanogen able to oxidize secondary alcohols. Archives of Microbiology 153:287-293.
4. Cadillo-Quiroz H, Yavitt JB, Zinder SH. 2009. *Methanosphaerula palustris* gen. nov., sp nov., a hydrogenotrophic methanogen isolated from a minerotrophic fen peatland. International Journal of Systematic and Evolutionary Microbiology 59:928-935.
5. Ferry JG, Smith PH, Wolfe R. 1974. *Methanospirillum*, a New Genus of Methanogenic Bacteria, and Characterization of *Methanospirillum hungatii* sp. nov. International Journal of Systematic and Evolutionary Microbiology 24:465-469.
6. Konig H, Stetter KO. 1982. Isolation and characterization of *Methanolobus-tindarius*, sp-nov, a coccoid methanogen growing only on methanol and methylamines. Zentralblatt Fur Bakteriologie Mikrobiologie Und Hygiene I Abteilung Originale C-Allgemeine Angewandte Und Okologische Mikrobiologie 3:478-490.
7. Lomans BP, Maas R, Luderer R, den Camp H, Pol A, van der Drift C, Vogels GD. 1999. Isolation and characterization of *Methanomethylovorans hollandica* gen. nov., sp nov., isolated from freshwater sediment, a methylotrophic methanogen able to grow on dimethyl sulfide and methanethiol. Applied and Environmental Microbiology 65:3641-3650.
8. Dridi B, Fardeau M-L, Ollivier B, Raoult D, Drancourt M. 2012. *Methanomassiliicoccus luminyensis* gen. nov., sp nov., a methanogenic archaeon isolated from human faeces. International Journal of Systematic and Evolutionary Microbiology 62:1902-1907.
9. Sowers KR, Ferry JG. 1983. Isolation and characterization of a methylotrophic marine methanogen, *Methanococcoides methylutens* gen. nov., sp. nov. Applied and environmental microbiology 45:684-690.
10. Ollivier BM, Mah RA, Garcia JL, Boone DR. 1986. Isolation and characterization of *Methanogenium bourgense* sp nov. International Journal of Systematic Bacteriology 36:297-301.
11. Miller TL, Wolin MJ, Demacario EC, Macario AJL. 1982. Isolation of *Methanobrevibacter smithii* from human feces. Applied and Environmental Microbiology 43:227-232.
